# Individualization of Treatment Improves the Survival of Children With High-Risk Solid Tumors: Comparative Patient Series Analysis in a Real-Life Scenario

**DOI:** 10.3389/fonc.2019.00644

**Published:** 2019-07-17

**Authors:** Michal Kyr, Kristyna Polaskova, Zuzana Kuttnerova, Tomas Merta, Jakub Neradil, Jitka Berkovcova, Ondrej Horky, Marta Jezova, Renata Veselska, Giannoula Lakka Klement, Dalibor Valik, Jaroslav Sterba

**Affiliations:** ^1^Department of Pediatric Oncology, University Hospital Brno and School of Medicine, Masaryk University, Brno, Czechia; ^2^International Clinical Research Centre, St. Anne's University Hospital Brno, Brno, Czechia; ^3^Laboratory of Tumor Biology, Department of Experimental Biology, School of Science, Masaryk University, Brno, Czechia; ^4^Laboratory of Molecular Pathology, Department of Oncological Pathology, Masaryk Memorial Cancer Institute, Brno, Czechia; ^5^Department of Pathology, University Hospital Brno and School of Medicine, Masaryk University, Brno, Czechia; ^6^CSTS Health Care Inc., Toronto, ON, Canada; ^7^Department of Laboratory Medicine, Masaryk Memorial Cancer Institute, Brno, Czechia

**Keywords:** cancer, children, personalized medicine, targeted therapy, comparative effectiveness research, clinical trials, metronomic

## Abstract

**Introduction:** The individualization of treatment is attractive, especially in children with high-risk cancer. In such a rare and very heterogeneous group of diseases, large population-based clinical randomized trials are not feasible without international collaboration. We therefore propose comparative patient series analysis in a real-life scenario.

**Methods:** Open cohort observational study, comparative analysis. Seventy patients with high-risk solid tumors diagnosed between 2003 and 2015 and in whom the treatment was individualized either empirically or based on biomarkers were analyzed. The heterogeneity of the cohort and repeated measurements were advantageously utilized to increase effective sample size using appropriate statistical tools.

**Results:** We demonstrated a beneficial effect of empirically given low-dose metronomic chemotherapy (HR 0.46 for relapses, *p* = 0.017) as well as various repurposed or targeted agents (HR 0.15 for deaths, *p* = 0.004) in a real-life scenario. However, targeted agents given on the basis of limited biological information were not beneficial.

**Conclusions:** Comparative patient series analysis provides institutional-level evidence for treatment individualization in high-risk pediatric malignancies. Our findings emphasize the need for a comprehensive, multi omics assessment of the tumor and the host as well whenever molecularly driven targeted therapies are being considered. Low-dose metronomic chemotherapy or local control of the disease may be a more rational option in situations where targeted treatment cannot be justified by robust evidence and comprehensive biological information. “Targeted drugs” may be given empirically with a realistic benefit expectation when based on robust rationale.

## Introduction

The success rate of cancer treatment in children has increased substantially in the past three decades (1). Despite progress, there are high-risk groups of children with cancer who do not respond to standard (maximum tolerated dose-based, MTD) treatments and continue to have poor outcomes. For this subset of patients with poor outcomes, the individualization of treatment is an emerging strategy. Such individualization may be understood as using not only targeted agents but also metronomic chemotherapies given beyond standard treatment options, if any, due to the specifically poor prognosis. This meaning of individualization i.e., customization of treatment beyond standard treatment using metronomic chemotherapy, repurposed, or targeted agents, is used throughout this paper. These approaches are well-described and employed in clinical practice (2–4).

Personalized treatment is a well-established concept aimed at optimizing patient therapy on the basis of the tumor and patient-specific biological profile. Pediatric malignancies are rare diseases in which specific alterations make personalization of therapy amenable. Classical population-based randomized clinical trials (RCT), considered a gold standard for evidence-based medicine, are incompatible with personalization of treatment for children with cancer. There is a need for the modernization of clinical trial methodologies, particularly the speed of the clinical trial process, and innovative designs (5, 6).

The aim of this study was to evaluate the efficacy of various individualized treatment approaches given either empirically or guided by biological information and to present an application of complex analytical solutions. High-risk malignancies in children form rare entities, which creates challenges in the evaluation of such small samples (7). Individualized alternatives to population-based clinical trials are the n-of-1 trials. Unfortunately, the use of the classical n-of-1 approach is not suitable in most pediatric patients with high-risk malignancies. We therefore suggest combining classical population-based and n-of-1 trials to form a series of n-of-1 trials. Existing statistical methodology enables handling specific statistical issues arising in such design. We present an application of such analytical tools, specifically extended Cox, frailty, and joint models, on a cohort of patients on individualized treatment. These tools are able to address repeated events, time varying covariates, known and unknown heterogeneity, and informative censoring problems, all of which are inherent to the individualized treatment settings. The key idea of increasing effective statistical power lies in combining rare entities to gather a larger heterogeneous sample, utilizing repeated measurements and appropriately addressing these factors in statistical analysis. This analysis was performed in a pragmatic real-life scenario that addresses more relevant clinical questions (6, 8). Comparative effectiveness research (9, 10) is an established concept and pragmatic observational studies render patient-centered real-life results that cannot be obtained through classical RCTs.

## Methods

### Sample Population

Children with relapsed and/or high-risk solid tumors for whom specific individualized treatment was recommended and who signed (or whose legal guardians signed) an informed consent were retrospectively or in a prospective manner enrolled in the data registry. Disease was defined as high-risk if the expected 5-years survival rate on standard therapies was <25%. Institutional review board approval for the study was obtained. Patients from the Pediatric Oncology Department, University Hospital Brno diagnosed between 2003 and 2015, and treated using individualized treatment strategy were analyzed. The retrospective cohort enrolled 11 patients treated until 2012 and 59 patients treated in 2013 and beyond constituted the prospective cohort.

### Treatment Assignment

Patients included in this cohort received standard first and/or subsequent lines of treatment regimens, if available, and individualized treatment. Altogether, the standard treatment regimens used MTD-based chemotherapy, surgery and/or radiation and originated from international pediatric oncology collaborative study groups. When standard treatment options were depleted or due to the high-risk nature of the disease, patients received individually assembled treatment consisting of metronomic chemotherapy, repurposed drugs and/or targeted agents such as antibodies or signal pathway inhibitors. At various time points of the treatment, a patient may have received molecular board consultation utilizing clinical and tumor biological data based on which a customized recommendation for specific targeted treatment was adopted. Thus, one can recognize the empirical period in which the treatments were given without knowledge based on biological studies, and the personalized (or targeted) period in which the tumor tissue studies opened the possibility to use a specific drug. Not all the drugs given in the targeted period needed to be given based on biological guidance (e.g., concomitant metronomic chemotherapy). Similarly, the selected targeted drugs did not need to be given during the whole targeted period, and even agents usually used as targeted could be given empirically (e.g., based on published data) in either of the periods. Thus, any of the drugs could be used in either of the period also as comedication, continuing medication from one period to the other, etc. It is necessary to understand that there were no protocol guidelines that would manage the choice of the treatment, time of its commencement or duration. Decision-making took place on daily clinical practice. Such a practice-based approach with intertwining treatments makes the perception of the design difficult compared to, e.g., simple two-arm-single-agent trial design. The simplification of our concept can be imagined as the patient timeline during which treatments were given to the patient based on best clinical judgement and, from a certain point, i.e., the tumor board consultation, the judgement could have been influenced by new biological information. Since that time, the targeted period has been considered.

In the absence of guidelines or protocols for individualized treatment strategies, the recommendations were based on best clinical practice arising from experienced clinical judgment, in-house protocols, published knowledge from either preclinical and/or clinical studies and biological studies comprising immunohistochemistry, TruSight Tumor 26 panel (Illumina Inc., San Diego, California, USA) for DNA level gene alterations and/or the phosphorylation profile of selected kinases and other signaling molecules (Human Phospho-RTK Array kit and Human Phospho-MAPK Array kit; R&D Systems, Minneapolis, MN, USA), if available. Modifications or new recommendations may have been made repeatedly for the same patient when toxicity, new clinical events, or new important information became available. Combinations of several drugs were used at the discretion of the attending oncologist or based on the consensus of molecular tumor board recommendation, which was in line with the philosophy of a multiple-hit strategy (3). In a number of cases, patients may have also received surgery or radiation with curative or palliative intend as a part of standard and/or individualized treatment.

The following treatment approaches were evaluated: (i) METRO—low-dose metronomically administered chemotherapeutics (Azacitidin, Cyclophosphamide, Etoposide, Methotrexate, Paclitaxel, Temozolomide, Vinblastine, Vinorelbin) (ii) REPUR—repurposed drugs (Arsen trioxide, Tretinoin, Isotretinoin, Celecoxib, Fenofibrate, Fluvastatin, Metformin, Miltefostin, Propranolol, Thalidomide, Valproate) (iii) INHIB—tyrosine kinase inhibitors, mTOR inhibitors, or monoclonal antibodies (Axitinib, Bevacizumab, Cabozatinib, Denosumab, Erlotinib, Ibrutinib, Idelalisib, Ipilimumab, Nimotuzumab, Nivolumab, Obinutuzumab, Pazopanib, Pembrolizumab, Regorafenib, Sirolimus, Sorafenib, Sunitinib, Temsirolimus, Trametinib, Vemurafenib) (iv) SURG/RT—local interventions, such as surgery with at least partial resection and local radiation.

There was no explicit control group. Each treatment approach was compared to the rest of the sample, which did not comprise the evaluated treatment. It is essential to realize that the smallest unit to be processed by the models is not the whole patient but the patient-day unit due to the time-varying covariates. Therefore, even when all patients received any of the evaluated treatment during some part of their disease history, there were also periods (i.e., patient-days) without any such treatment at all in each of the patients. Specifically, periods with an evaluated treatment approach were compared to periods without a particular treatment approach. By the nature of the design, these control periods comprised periods with different treatment approaches other than the evaluated periods, periods without any of the evaluated treatments and the control periods came from both different (between-) and the same (within-comparison) patients. Thus, these periods formed a control background to which each evaluated treatment approach was compared. The control background was not exactly the same for each evaluated treatment. We hypothesized that the differences between control backgrounds were negligible compared to effects due to specific treatment that was evaluated due to random heterogeneity in the sample. This assumption was checked by comparing the control backgrounds.

### Statistical Methodology

Detailed parameterized data were recorded in a relational SQL-based database allowing for time-varying covariate data structure and enabling data retrieval in a format needed for creating risk sets based on specific survival models.

The effects of different treatment strategies in patients treated with individualized therapeutic approaches were evaluated using various extensions of Cox models (variance corrected or frailty models) or joint models to account for time varying treatments, repeated data, subject heterogeneity, event dependence, and informative censoring. A detailed explanation of all models used is given elsewhere (11–18). We encourage readers to review a brief summary of the method used, which is available in the Supplementary Material, to better appreciate the methodical background for interpretation. Specifications of the models used are given in Table 1. Treatment approaches were analyzed in multivariate models and adjusted for possible bias.

**Table 1:** Specification of survival models.

**Variance corrected models**	
Wei, Lin, and Weissfeld (WLW)	$h_{ik}\left(t\right)=h_{0k}\left(t\right)e^{x_{ik}\beta }$
Prentice, Williams, and Peterson (PWP)	$h_{ik}\left(t\right)=h_{0k}\left(t\right)e^{x_{ik}\beta }$
Andersen and Gill (AG)	$h_{ik}\left(t\right)=h_{0}\left(t\right)e^{x_{ik}\beta }$
**Frailty models**	
Conditional frailty	$h_{ik}\left(t\right)={u_{i}h}_{0k}\left(t\right)e^{x_{ik}\beta }$
Shared frailty	$h_{ik}\left(t\right)={u_{i}h}_{0}\left(t\right)e^{x_{ik}\beta }$
**General joint model**	
Recurrent event Terminal event	$\left\{\begin{array}{c} r_{ik}\left(t\right)={u_{i}r}_{0}\left(t\right)e^{x_{Rik}\beta_{R}}\\ \lambda_{i}\left(t\right)={u_{i}^{\alpha }\lambda }_{0}\left(t\right)e^{x_{Ti}\beta_{T}} \end{array}\right\}$

*Where variance of u_i_ var(u_i_) = θ*.

The events of interest were progressions or relapses, evaluated by common RECIST criteria, deaths, or a combined measure of event-free survival (EFS) depending on a specific model. Analyses were performed using R Core Team software (19) version 3.3.3, coxph (20) and frailtyPenal (21–23) functions.

### Sample Size Justification

This study was a registry-based analysis where treatment allocations were not randomized and were not blinded to investigators as they followed the best clinical practice and best patient interest, thus, no sample size analysis was performed in advance. We considered a rule of thumb of at least 10 events per variable (24) for the classical Cox model to be valid when building our models. Although we consider the commonly accepted significance level α = 5% for interpretation of our results we may also consider the trends and effects of clinical relevance.

## Results

**Figure 1:**
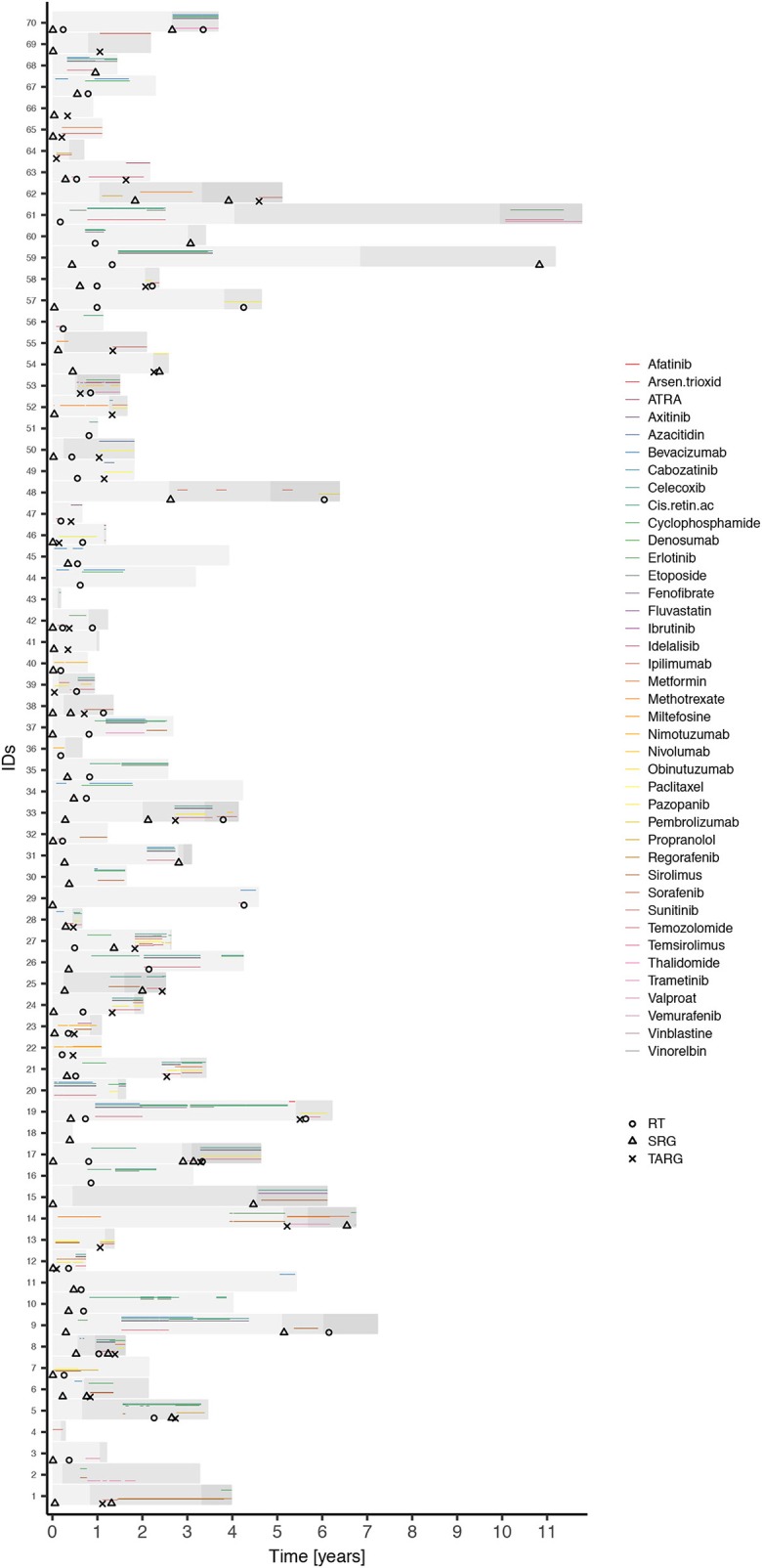
Schematic overview of individual treatment plans. Figure schematically shows the treatment plans of individual patients. Three shades of gray bars represent periods to the first, second, and third event, respectively, within a patient. Circles, triangles, and crosses indicate irradiation (RT), surgery (SRG), and biologically guided approach commencement (TARG), respectively. Rainbow-like set of active agents is presented to illustrate the complexity of treatment plans in patients with individualized therapeutic approaches. Multiple combinations of different drugs commencing at different time points and changing within an individual patient create unique cases rather than similar groups.

**Figure 2:**
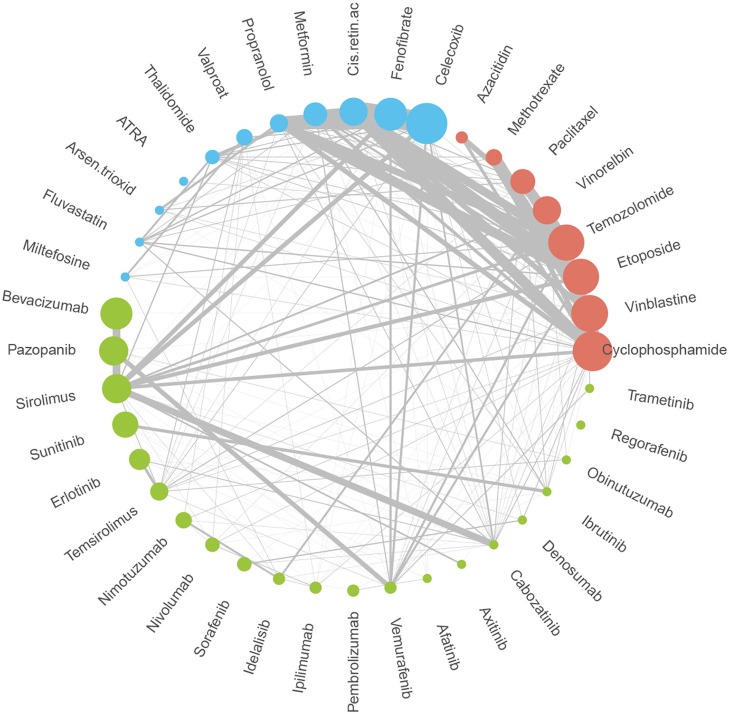
Drugs and combinations utilized during individualized treatment. Circle size is proportional to the number of patients receiving the drug during any phase of the treatment. Line thickness is proportional to patient-days using a specific combination of a drug in the sample during the treatment.

**Figure 3:**
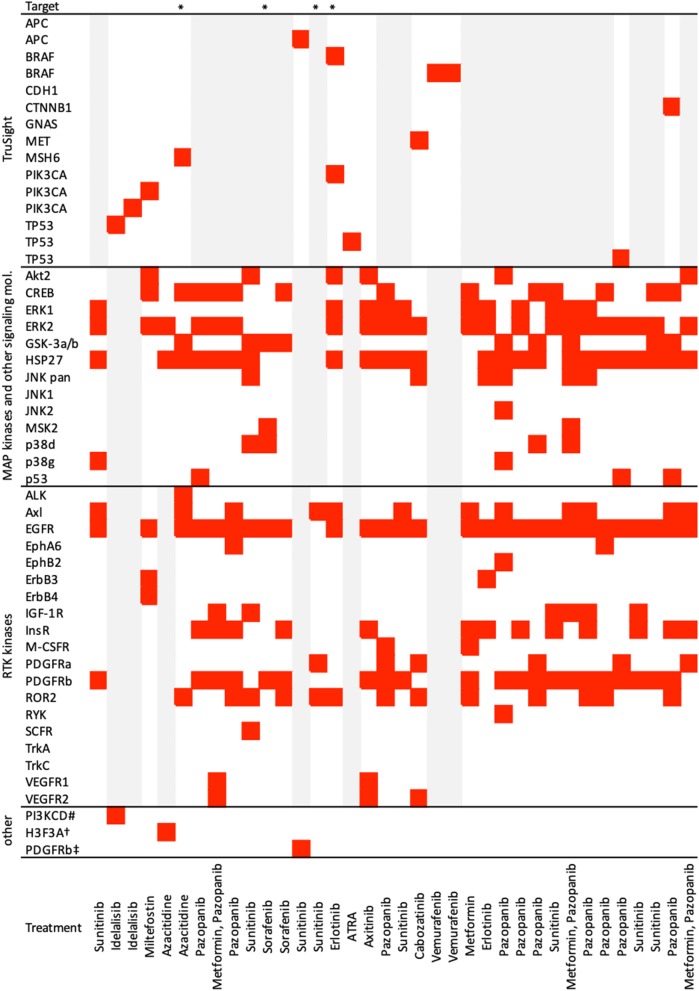
Results of biological examinations and treatment summary. Figure shows findings of biological tissue examination and drugs selected in patients on targeted (TARG) management. Other concomitant medications are not indicated here. *Treatment classified as targeted retrospectively, sensitivity analysis performed; ^#^germinal mutation, c.935C>G, Sanger seq.; ^†^c.83A>T/p.K28M, Whole exome sequencing; ^‡^immunohistochemistry; gray bars—not done.

A total of 70 patients (36 males and 34 females, mean age 7.2 ± 5.6 years) were enrolled in the analysis. Sample characteristics are summarized in Table 2. Patients on the individualized treatment approach were followed for a median of 26 months. During this time, the patients experienced 67 recurrent events in total (relapses or progressions) and 20 terminal events (deaths). A biologically guided (personalized) approach was applied in 36 patients. A substantially lower number of patients was available for the evaluation of each subsequent event. The third event group comprised four patients with even a fourth event. Individual treatment schedules are schematically given in Figure 1. Figure 2 illustrates the drug combinations. These figures are intended to roughly illustrate the complexity of the treatment. Figure 3 shows the findings of the biological examination of tumor tissues and specific targeted drugs selected in patients for whom the targeted management was applied. Other concomitant treatments given to these patients are not indicated in this scheme.

**Table 2:** Sample characteristics.

	**Event rank 1 (baseline)**	**Event rank 2**	Event rank 3^*^
	***n* = 70**	***n* = 44**	***n* = 17**
**Diagnoses, No. patients**			
Soft tissue and other extraosseous sarcomas	24	16	4
CNS and miscellaneous intracranial and intraspinal neoplasms	22	12	4
Malignant bone tumors	9	8	5
Neuroblastoma and other peripheral nervous cell tumors	8	2	0
Hepatic tumors	3	2	0
Malignant melanomas	2	2	2
Lymphomas and reticuloendothelial neoplasms	1	1	1
Renal tumors	1	1	1
**Demographics**			
M/F, *N*	36/34	22/22	7/10
Age; mean ±*SD*, years	7.2 ± 5.6	7.5 ± 5.7	7.6 ± 4.7
Personalized (biologically guided) approach, N	17	24	9
**Treatment approaches (empirical/targeted period)**, ***N***			
METRO	35/15	13/15	5/7
REPUR	20/10	9/11	6/7
INHIB	25/15	13/19	7/7
SURG/RT	57/14	16/11	6/7
**Events**, ***N*** **(%)**			
Progression/relapses	46 (66%)	16 (36%)	5 (30%)
Deaths	4 (6%)	13 (30%)	3 (18%)
Overall survival; median, years	7.2		
Follow-up; median (min—max), months	26 (2–141)		

* *Comprises 4 patients with a fourth event*.

**Figure 4:**
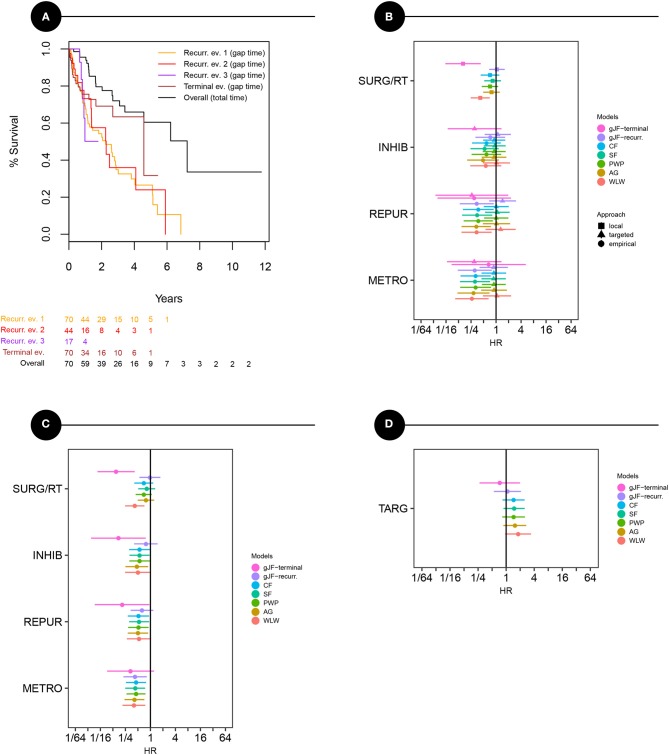
Survival models of specific events and treatment effect estimates overview. **(A)** Kaplan-Maier curves representing survival times for each recurrent event (yellow, red, purple lines) and terminal event (brown line) as interevent (gap) times, and overall survival curve (black). **(B–D)** Effects estimates and confidence limits of different treatment modalities in bias-adjusted models: **(B)** pragmatic view representing raw effects of treatment groups separately in targeted and non-targeted period, **(C)** conditional, quasi-randomized view (adjusted for targeted/non-targeted period) representing a pooled treatment effects across periods, **(D)** effect of targeted (biologically guided) management itself. Models: marginal – Wei, Lin, and Weissfeld, AG – Andersen and Gill, PWP-GT – Prentice, Williams, and Peterson model in gap time, SF – shared frailty, CF – conditional frailty, gJF – general joint frailty model with effect for recurrent (recur) and terminal (term) event. METRO – metronomically given low-dose chemotherapeutics, REPUR – repurposed drugs, INHIB – signal pathway inhibitors/mTOR inhibitors/antibodies, SURG/RT – local treatment (surgery and/or irradiation). To the left from the midline favors treatment (survival improvement).

Overall Kaplan-Meier curves for recurrent and terminal events were calculated as interevent (or gap) times and are given in Figure 4A. An overall survival curve (OS) calculated as the total time elapsed from an initial diagnosis was also plotted for comparison. All three recurrent events seem close to each other suggesting no major differences in survival times. On the other hand, there is improvement indicated in time to death beyond surviving a second year compared to previous events. The overall median survival time from the initial diagnosis was 7.2 years.

Effects estimates of bias-adjusted models are displayed in Figures 4B–D. The only strong observed covariate (rendering bias if omitted) associated with outcome was calendar time, which is not surprising due to the long study period covered. Therefore, we adjusted all models for this covariate. Figure 4B shows the model results, where treatment effects were modeled and shown separately for the empirical and the personalized period and represents a pragmatic view. It is obvious that treatments were more effective when given during the empirical period. Most beneficial effects on EFS were metronomically given low-dose classical chemotherapeutics. No obvious treatment benefit was noted in the personalized period for either of the treatments. Local treatment was shown to be beneficial for prolonging survival during the terminal event, which could be identified only using joint models.

The models shown in Figure 4C were adjusted for empirical/personalized periods, and the estimates represent pooled quasi-randomized treatment effects. Based on this analysis, we can see that all three drug groups are beneficial, with the most evident effect on EFS for metronomically given low dose chemotherapeutics. Repurposed drug or inhibitor effects on EFS were close to significance border. On the other hand, the results showed a significant and strong beneficial effect on overall survival during the terminal event. The beneficial effect of local treatment was noted only with respect to overall survival without affecting EFS or recurrences.

Figure 4D shows estimates of the personalized approach itself, i.e., effect of potentially guiding biological information, showing no benefit on EFS.

Finally, we evaluated all treatment strategies in multivariate models. The final models generated are given in Table 3. A conditional frailty model for combined outcome (EFS) was used as the most appropriate. Metronomically given low-dose chemotherapy was the only significantly beneficial treatment that halved the risk (HR 0.45, *p* = 0.005) with respect to EFS. To evaluate jointly modeled relapses and deaths, general frailty model was used. Metronomically given chemotherapy remained the only significantly beneficial effect on relapses (HR 0.46, *p* = 0.017) in this model. However, repurposed drugs or inhibitors/antibodies significantly improved survival (HR 0.15, *p* = 0.004) during the terminal event (death) and the local treatment (HR 0.16, *p* = 0.001) also improved survival during the terminal event. Notably, the size of the metronomic treatment effect on deaths was similar to that on EFS or relapses but did not reach conventional statistical significance because the number of deaths was markedly lower than the number of repeated events. This effect is also evident from wide confidence limits in Figures 4B–D for that particular model (general joint frailty model).

**Table 3:** Parameter estimates of final multivariate models.

**Model**	**Treatment**	**HR**	***p***
Conditional frailty (EFS combined)	METRO	0.45	0.005
General joint frailty (relapses)	METRO	0.46	0.017
General joint frailty (deaths)	REPUR/INHIB	0.15	0.004
	SURG/RT	0.16	0.001

*Final bias-adjusted models from multivariate analysis represented by the most appropriate model (conditional frailty model for EFS and general joint frailty model for joint modeling of relapses and deaths)*.

## Discussion

The evaluation of individualized therapies in children with high-risk cancer is difficult when using traditional approaches, such as large single-agent/approach RCTs. No matter how grateful we are for such clear evidence of treatment benefit, performing RCTs require international collaboration, and is redeemed by a time-consuming process. Based on recent experience, it may take 12 years to yield a single conclusion (25). In most pediatric cancer cases, traditional large population-based approaches may not be possible at all and contradict the driving philosophy of personalized medicine. Lindsey and Lambert wrote an excellent examination of the marginal (population-based) vs. conditional (patient-centered) inferences (26) which may help understand the limitations of classical population-based trials.

This was a pragmatic observational study dealing with real-world data, where various aspects of the complex data were addressed using appropriate statistical tools. These techniques have already been described long ago and applied in various research areas outside medicine (14). As shown in simulation studies (15, 27) for situations where event dependence and heterogeneity arise simultaneously from the same data, the conditional frailty model appears to be the most appropriate. However, in our study, we could not observe any substantial difference in treatment effects for conditional frailty models. This finding suggests that unmeasured heterogeneity between patients or event order did not substantially affect our models. Nevertheless, we still recommend using stratified and/or frailty models, at least as a part of the analysis process because it might become an important factor with different data and, more importantly, these models enable conditional (patient-centered) inferences. In general, similar effects were observed using different Cox-extension models or frailty models. The joint frailty models, however, showed different effects for recurrent and terminal events, as was obvious for repurposed drugs and inhibitors as well as local treatments. Such a fact, if ignored in conventional analysis, might lead to incorrect conclusions.

A similar EFS curve found for different ranks of events (Figure 4A) is an interesting finding. This suggests that regardless of the treatment strategy selected in the individualized scenario, the times to subsequent events were roughly similar without shortened survival that would be normally anticipated in the course of progressive malignant disease. This similar event-rank risk may also explain the small importance of rank stratification in the models in our data.

The efficacy of metronomically given low-dose chemotherapy, particularly cyclophosphamide, or vinblastine, has already been described (28–30). Here, we were able to identify their favorable effects in a customized observational pragmatic setting. A number of mechanisms of action are considered responsible for the efficacy of metronomic therapies. Antineoplastic, immunomodulatory, antiangiogenic, and tumor microenvironmental activity are reported most often (2).

Our results suggest similar effects of repurposed drugs and various signal pathway inhibitors or antibodies. Their beneficial effect on overall survival during the last (terminal) event was evident while we could not conclude that for recurrences. This results suggests that patients may live longer even though the disease itself does not regress when evaluated using conventional imaging studies and RECIST criteria. These observations are also complemented by our clinical experience in the number of patients who repeatedly reported improved well-being while on the medication, although we had no objective evidence of disease burden reduction or the patients even slowly progressed. The mechanism of action of numerous repurposed drugs or kinase inhibitors is far from cytotoxic, and stable disease may reflect only their different functional impact in cancer treatment. We face different mechanisms of action with new drugs that result in different disease behaviors. It thus raises the question of whether classical response criteria are optimal for personalized medicine.

Similar but stronger observations were found for local control of the disease. Local treatment did not influence the natural course of disease (reflected by recurrences) but may significantly improve survival. Providing additional time with relatively good quality of life may be an acceptable goal to refractory patients and, more importantly, may open a window of opportunity for other treatment modalities such as targeted therapies and immune therapies.

Utilizing the available knowledge arising from tissue analyses for biological guidance of the treatment we had at the time of early implementation of targeted therapies (in the present analysis) did not prove beneficial. The biological analyses were limited to kinase phosphorylation status and/or TruSight® Illumina NGS panel in only a few patients. Although these technologies may be useful in certain cases (31), they may not necessarily be sufficient for most analyzed patients. Several candidate alterations could be identified in most patients, but this fact does not warrant that an effective targeted therapy had to be utilized. The relevance and actionability of detected alteration are an issue (32). Specifically, if either only limited biological information is available or a detected alteration is assumed principal or driving in tumorigenesis but is actually not, it is not surprising that guidance of the targeted therapy having been based on such insufficient or irrelevant evidence was not beneficial. On the other hand, no effective drug can be available at all for a real driving alteration identified. We may also hypothesize that alterations mainly found among kinase phosphorylation profiles only indicated broad dysregulation of signal pathways. Several targeting agents might have been necessary in such situations to prove effective. Combined therapy is potentially more effective as is in accordance with multiple-hit philosophy (3) but also challenging due to unknown toxicity and interactions of novel agents. Molecular oncology board recommendations were based on very limited information compared to the currently utilized technologies, which include modern whole genome/exome sequencing and transcription analyses. A better understanding and correct interpretation of multi-omics data might provide more promising results in the near future.

The most surprising fact, however, was that treatment was more beneficial when given during the empirical period. There might be residual bias due to a combination of small factors that could not be accounted for in a more complex multivariate model and summed up to form more and less risk periods and possibly being responsible for these results in part. However, we think that this observation could be explained through Bayesian reasoning as follows. Tumor board recommendations are based on various information, such as published data (prior evidence) and biological evaluation (new data). A new recommendation is then synthetized (posterior information). Philosophy of personalized medicine guides to base the evidence mainly on individual biological data. However, if based on limited or irrelevant information, it might divert our focus from, e.g., robust published evidence, although population-based, to less reliable or irrelevant individual data. Thus, the effect could be viewed as overweighting less reliable new data over underweighted prior robust evidence. Therefore, we strongly encourage the use of population-based evidence together with highly personalized approaches and comprehensive biological evaluation. Encouraging results of “personalized drugs” used on empirical grounds offers possibilities for patients in whom no target could be found.

There are several limitations that need to be discussed. This study was an observational, comparative-effectiveness study in which various sources of bias may have been introduced. Indication bias or unbalanced groups may be the most important factors. The long period covered in the analysis might have introduced inconsistencies in treatment indication strategies resulting in mentioned unbalances or bias. On the other hand, various factors were accounted for through the statistical tools used. Specifically, we believe that random effects in frailty models, event rank stratification and adjustment to calendar time in multivariate models could address most of the bias. Another question was the expected treatment effect onset and its duration after treatment initiation and discontinuation. We did not presume any specific time pattern of the treatment effects. Different coding of time-varying factors in the models could answer such specific questions. Furthermore, the interpretation of the results in the observational comparative setting needs to be adapted to the study design and in-house protocols. For example, we should regard the effects of the evaluated therapies in the context of concomitant or alternative treatment options being or having been given to our patients. Similarly, drug interactions or comedication effects could not be addressed because protocol-based same combinations or, on the other hand, individual single-cases of drug combinations arise. Thus, we cannot answer questions, such as which drug was most effective in which situation. However, the aim of the analysis was not the efficacy of a single drug but rather the treatment principle/modality under an individualized approach. There was no explicit control group in our data set, but we hypothesized that the sample itself rendered comparable control backgrounds to which the specific evaluated therapy of interest was compared. This assumption was also checked using the models.

We did not specifically address toxicity in this study. However, individualized management of a patient comprises standard assessment of toxicity and adjusting the treatment appropriately, and most patients were managed on an outpatient basis only.

## Conclusions

Comparative patient series analysis provides institutional-level evidence for individualization of treatment in children with high-risk malignancies. Targeted treatment based on limited biological information is not beneficial for patients, which stresses the need for comprehensive multi-omics biological studies. Low-dose metronomic chemotherapy or local control of the disease may be a more rational option where targeted treatment cannot be justified by robust evidence and comprehensive information regarding tumor and the host biology. On the other hand, “targeted drugs” may be given empirically with a realistic benefit expectation when based on a robust rationale.

## Data Availability

The datasets for this study will not be made publicly available because general provision of data is not in accordance with our institutional policy. We handle data of rare entities that may be at risk of identification even when anonymized. A journal representative may be invited for control purposes and the data sets may be temporarily provided on site and under the supervision of the institution.

## Ethics Statement

All subjects or their legal guardians gave written informed consent in accordance with the Declaration of Helsinki. The study was approved by the Institutional review board of University hospital Brno.

## Author Contributions

MK designed the study, designed and created the registry SQL database, performed statistical analyses, and wrote the manuscript. KP evaluated patient records, performed data registry, and wrote the manuscript. ZK and TM evaluated patient records and performed data registry. JN, RV, JB, OH, and MJ performed biological samples analyses. GK and DV supervised and wrote the manuscript. JS conceived and supervised the project and wrote the manuscript.

### Conflict of Interest Statement

GK was employed by CSTS Health Care Inc., Toronto, Ontario, Canada. The remaining authors declare that the research was conducted in the absence of any commercial or financial relationships that could be construed as a potential conflict of interest.
