# Supplementary material for: Individualization of Treatment Improves the Survival of Children With High-Risk Solid Tumors: Comparative Patient Series Analysis in a Real-Life Scenario
Source: Front Oncol. 2019 Jul 17;9:644. doi: 10.3389/fonc.2019.00644 (PMC6650566; doi:10.3389/fonc.2019.00644)
Supplement: Supplementary file 1 [file Table_1.pdf]

Supplementary material

Review of statistical methodology

The models used are of three types: i) variance-corrected models, ii) frailty models and iii) joint models.

The variance-corrected models account for unobserved heterogeneity through robust errors and sometimes stratification. The Wei, Lin, and Weissfeld (WLW) model is a marginal model with a traditional competing risk problem in which inferences for each event do not take any other event into account. This model is not generally recommended in most analyses with repeated events, but we included this model for comparison. The Andersen and Gill (AG) model is the simplest variance-corrected model. Repeated events are accounted for through robust errors, and events for the same subjects are considered independent. This model, however, restricts the baseline hazard for all events to be the same. The Prentice, Williams, and Peterson (PWP) model is the most flexible of the group. For this model, explicit ordering of sequential events is characteristic. Specifically, k-th event occurrence for an individual is conditioned on previous k-1-th events having occurred in that subject. Moreover, this model is stratified on an event rank allowing for different baseline hazards for different event ranks. This model seems intuitively the most appropriate choice out of the variance-corrected model group for our data. We considered the gap-time (interevent) schedule in this model by counting the time to a next event since a previous event.

Frailty models are a group of models in which the heterogeneity is modeled through random effects called “frailties” on each subject with an assumption of the distribution of the frailties. In a shared frailty model (the simpler of the group), there is a random effect for each subject that is shared over time by the respective subject. Conditional frailty models, on the other hand, allow for specific baseline risk on different occasions through stratification similar to the PWP model. For both frailty models, the gap-time (interevent) schedule was also considered.

For the above models, we used a composite outcome measure (event free survival, EFS) so that an event was considered either relapse/progression or death.

Joint models are stand-alone models, where informative censoring or different event type dependence is accounted for through joint modeling of the respective event types. It enables the distinction of the different effects of a factor (e.g. treatment) specifically on relapse/progression (recurrent event) or death (terminal event) without necessity of censoring one type of event or creating a composite measure. Censoring one type of event is not recommended for dependent events such as repeated within-subject observations. We used the

joint frailty model in which two types of events (relapse/progression and death) were modeled with shared frailties.
